# Chloroform Anæsthesia

**Published:** 1898-06-25

**Authors:** 


### CHLOROFORM ANÆSTHESIA.

Writing about the production of chloroform anæsthesia, Surgeon Lieutenant-Colonel Lawrie, of Hyderabad, says: "In Hyderabad chloroform is given by students in strict accordance with Syme's principles, the heart is ignored, and we have no deaths. In England the teaching of anæsthetics is in the hands of professional anæsthetists, who 'funk' the heart, and deaths from chloroform take place, according to Leonard Hill, by the dozen, and, according to Roger Williams, no less than once in every 1,236 inhalations. The deaths under anæsthetics in England represent an appalling picture of the incapacity of the medical profession as regards the administration of these drugs. This incapacity is altogether due to the absolute want of training of English students in the art of chloroform administration on Syme's principles, which have been established on a scientific basis by the Hyberabad Commission." This is an old indictment and an old assumption, and we have very little doubt that both are wrong. Certainly the "professional anæsthetists" here spoken of with such scorn no more neglect the respiration than Colonel Lawrie does, and as to the inhalers used in this country, some of them leave quite as free an air way as a Hyderabad cone does. The question is to a large extent one of dose, and although we do not approve of the open or slap-dash method, we are to this extent in sympathy with Colonel Lawrie that we do think that, if that is to be accepted as the proper way of administering chloroform, the method ought to be properly taught, and that the importance of plenty of air should be as much insisted on as that of plenty of chloroform. It is here we fancy that the young student often fails, and that the inexperienced practitioner gets into difficulties. The open method even with air is uncertain, but the open method without plenty of fresh air is absolutely dangerous. But then air and plenty of it was what Syme taught, and what is still taught, even in benighted England. Suffocative anæsthesia is the resource of the inexperienced. Whether it would not be better still to teach that chloroform should be so administered that the dose given can be known and can be regulated, and by an apparatus which ensures that in proportion as the respiration is retarded or interfered with the chloroform may also be withheld, is another question. This will doubtless come in time, for even the advocates of the open method must sometime see that such an apparatus is really an "open" one, more open, perhaps, than a Hyderabad cone, and that by its means the chloroform can be adjusted precisely to the respirations. In the meantime there can be no doubt that Colonel Lawrie is wrong in his estimate of English "professional anæsthetists," as he probably is also in his ideas as to the relation between circulatory and respiratory failure, although as to this we speak with some reserve.

